# Oral bowel cleansers and ischemic colitis risk: A real-world disproportionality analysis

**DOI:** 10.1371/journal.pone.0332345

**Published:** 2025-09-29

**Authors:** Min Luo, Wenyu Li, Xuehong Wang, Yuqian Zhou

**Affiliations:** Gastroenterology Department of the Second Xiangya Hospital, The Central South University, Changsha, Hunan, China; Dalin Tzu Chi Hospital, Buddhist Tzu Chi Medical Foundation, TAIWAN

## Abstract

**Background:**

Ischemic colitis (IC) is a serious but underrecognized complication potentially associated with bowel preparation. While previous studies have reported sporadic cases, the true frequency and drug-specific associations remain unclear. This study evaluates the association between oral bowel cleansers and IC using real-world pharmacovigilance data.

**Methods:**

We conducted a disproportionality analysis using 20 years of data (2004–2024) from the FDA Adverse Event Reporting System (FAERS). IC cases linked to bisacodyl, polyethylene glycol (PEG), and oral sulfate solution (OSS) were identified. Multivariate logistic regression was applied to explore factors associated with IC and serious clinical outcomes.

**Results:**

Among 43,958 adverse event reports related to bowel cleansers, 75 cases of IC were identified. Bisacodyl showed the strongest disproportionality signal for IC (reporting odds ratio (ROR) = 237.25), with a reporting proportion of 7.9%, followed by PEG (ROR = 2.18) and OSS (ROR = 3.64). Older age (≥70 years) and cardiovascular comorbidities were associated with more severe outcomes, such as hospitalization and death. Notably, reports of IC associated with PEG included six fatal and three life-threatening events.

**Conclusions:**

To our knowledge, this is one of the largest pharmacovigilance analyses exploring ischemic colitis associated with bowel preparation agents. The findings raise concerns about the presumed safety of PEG and reveal a strong disproportionality signal for bisacodyl. These results highlight the need for individualized bowel preparation strategies, especially in elderly patients with comorbidities.

## 1. Introduction

Ischemic colitis (IC) is an inflammatory condition associated with insufficient colonic blood supply. While its overall reporting portion is estimated at 17.7–22.9 per 100,000 individuals annually, its prevalence is significantly higher among the elderly and those with cardiovascular comorbidities [1–3].

Bowel cleansers are commonly administered before colonoscopy, surgery, and imaging procedures to ensure optimal examination conditions. IC as a complication of bowel preparation for colonoscopy has been increasingly recognized [4], with several case reports and small-scale studies implicating sodium phosphate, bisacodyl, and polyethylene glycol (PEG)-based regimens [5,6]. However, large-scale drug-specific association assessments remain scarce, leading to potential underestimation of IC risk in clinical practice.

Previous studies have primarily focused on single-center retrospective analyses [4]. However, no large-scale real-world pharmacovigilance studies have quantified the disproportionate association among different bowel cleansers. To address this gap, our study utilizes FAERS data spanning 20 years (2004–2024) to conduct a disproportionality analysis, systematically evaluating the IC association related with bisacodyl, PEG, and oral sulfate solution (OSS). By assessing patient demographics, drug-specific IC association, and severe outcomes, we aim to provide the most comprehensive association stratification to date. These findings are critical for optimizing safer bowel preparation regimens, particularly in high-association elderly populations.

## 2. Materials and methods

### 2.1 Data extraction and processing

The ASCII (The American Standard Code for Information Interchange) report files from January 2004 to September 2024 (publicly accessible at FAERS Latest Quarterly Data Files) were downloaded and analyzed using Microsoft Excel 2021 and R Studio (v2024.12.0) for data extraction, harmonization, and statistical modeling. This comprehensive pharmacovigilance platform integrates seven core data modules: (1) demographic and administrative information (DEMO), (2) drug exposure details (DRUG), (3) adverse event documentation (REAC), (4) clinical outcome tracking (OUTC), (5) reporting source information (RPSR), (6) treatment duration parameters (THER), and (7) indication-specific medication use (INDI).

**Fig 1:**
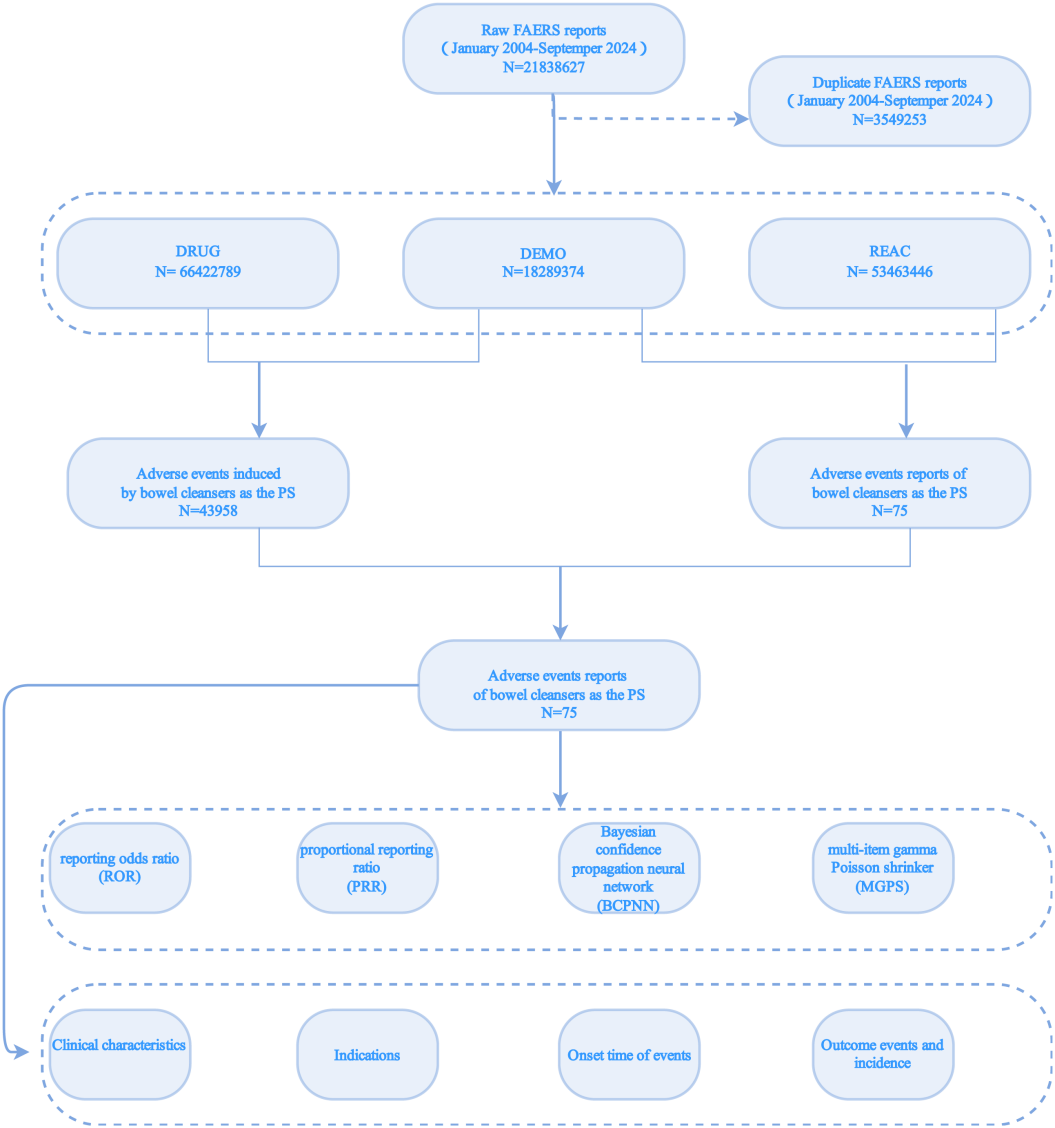
Flowchart.

The data standardization procedures adhered to FDA guidelines for duplicate removal and quality control. Key identifier fields (PRIMARYID, CASEID, FDA_DT) were extracted from the demographic dataset and prioritized as follows: (1) Records with identical CASEIDs were filtered to retain those with the most recent FDA_DT timestamp; (2) If multiple entries had matching CASEID-FDA_DT combinations, the record with the highest PRIMARYID value was preserved. The complete methodological workflow is illustrated in Fig 1.

### 2.2 Study population selection criteria

Pharmacological agents were categorized based on their potential etiological contribution to adverse events (AEs), comprising four distinct classifications: primary suspect (PS), secondary suspect (SS), concomitant (C), and interacting medications (I). In the initial analysis, only cases involving primary suspect drugs were included due to their stronger pharmacovigilance signal. The remaining classifications were excluded from primary analysis due to their diminished causal probability and increased uncertainty in adverse event attribution, though concomitant medications were retained for subsequent stratified analyses.

Drug identification was performed through systematic nomenclature verification, utilizing both brand and generic names cross-referenced against authoritative sources including with data of Drugs@FDA, Drugbank [7,8] and PubMed MeSH terminology. The Bisacodyl group comprised bowel preparation regimens containing bisacodyl alone or in combination with PEG or OSS. The PEG group primarily consists of combinations of PEG with various electrolyte formulations, but excludes bowel preparation agents containing bisacodyl or OSS. The OSS group includes bowel preparation agents formulated with oral sulfates, sodium picosulfate, etc., and excludes regimens containing bisacodyl or PEG components. In our search of the FAERS database, we did not identify any reports of sodium phosphate-based regimens associated with IC; thus, the corresponding content was not included in the results. The detail names of drugs were listed in S1 Table.

Cases were selected only from reports listing IC as the primary adverse event. In the FAERS database, AEs are coded using the Medical Dictionary for Regulatory Activities (MedDRA, version 26.2). Preferred terms (PTs) are used to describe specific AEs. System Organ Classes (SOCs), the highest level in MedDRA, categorize medical conditions by organ system or function. In this study, the SOC code is 10017947, with detailed PT codes listed in S2 Table.

### 2.3 Disproportionality analysis

The disproportionality analysis was conducted using a 2 × 2 contingency tables (Table 1) [9], applying four distinct computational algorithms: the Reporting Odds Ratio (ROR) [10], Proportional Reporting Ratio (PRR) [11], Bayesian Confidence Propagation Neural Network (BPCNN) [12], and Multi-item Gamma Poisson Shrinker (MGPS) [13].

**Table 1:** 2 × 2 contingency table for disproportionality analysis.

	Target AEs	Non-target AEs
Target drugs	a	b
Non-target drugs	c	d

AEs, adverse events. a: Reports with both target drug and target AE. b: Target drug reports with non-target AEs. c: Non-target drug reports with target AEs. d: Reports without target drug or target AE.

Mathematical formulations and threshold criteria for signal detection are comprehensively outlined in Table 2 [9]. For all metrics (ROR, PRR, BCPNN, MGPS), higher values generally indicate a stronger association between a drug and adverse event [14].

**Table 2:** Methods of disproportionality analysis and signal detection criteria.

Methods	Calculation formula	Criteria
ROR	ROR = $\frac{\left(\text{a}/\text{c}\right)}{\left(\text{c}/\text{d}\right)}$	a ≥ 3 and 95% CI (low limit) >1
	SE (lnROR)=$\sqrt{(\frac{\text{1}}{\text{a}}+\frac{\text{1}}{\text{b}}+\frac{\text{1}}{\text{c}}+\frac{\text{1}}{\text{d}})}$	
	95%CI=${\text{e}}^{\text{ln}\left(\text{ROR}\right)\pm \text{1}.\text{96}\sqrt{(\frac{\text{1}}{\text{a}}+\frac{\text{1}}{\text{b}}+\frac{\text{1}}{\text{c}}+\frac{\text{1}}{\text{d}})}}$	
PRR	PRR = $\frac{\frac{\text{a}}{(\text{a}+\text{b})}}{\frac{\text{c}}{(\text{c}+\text{d})}}$	a ≥ 3 and 95% CI (low limit) >1
	SE (lnPRR) = $\sqrt{(\frac{\text{1}}{\text{a}}-\frac{\text{1}}{\text{a}+\text{b}}+\frac{\text{1}}{\text{c}}-\frac{\text{1}}{\text{c}+\text{d}})\;}$	
	95% CI=${\text{e}}^{\text{ln}\left(\text{PRR}\right)\pm \text{1}.\text{96}\sqrt{(\frac{\text{1}}{\text{a}}-\frac{\text{1}}{\text{a}+\text{b}}+\frac{\text{1}}{\text{c}}-\frac{\text{1}}{\text{c}+\text{d}})}}$	
BCPNN	IC* =${\text{log}}_{\text{2}}\frac{\text{a}(\text{a}+\text{b}+\text{c}+\text{d})}{(\text{a}+\text{b})(\text{a}+\text{c})}$	1. No Signal (-): IC*_025_ ≤ 0 2. Low Signal (+): 0 < IC*_025_ ≤ 1.5 3. Medium Signal (++): 1.5 < IC*_025_ ≤ 3 4. High Signal (+++): IC*_025_ > 3
	E(IC*) = ${\text{log}}_{\text{2}}\frac{\left(\text{a}+\gamma \text{11}\mathrm{\backslash rightleft}(\text{a}+\text{b}+\text{c}+\text{d}+\alpha \right)\left(\text{a}+\text{b}+\text{c}+\text{d}+\beta \right)}{\left(\text{a}+\text{b}+\text{c}+\text{d}+\gamma \right)\left(\text{a}+\text{b}+\alpha \text{1}\right)\left(\text{a}+\text{c}+\beta \text{1}\right)}$	
	V(IC*) =$\frac{\text{1}}{{\left(\text{ln2}\right)}^{\text{2}}}\left\{\left[\frac{\left(\text{a}+\text{b}+\text{c}+\text{d}\right)-\text{a}+\gamma -\gamma \text{11}}{(\text{a}+\gamma \text{11})(\text{1}+\text{a}+\text{b}+\text{c}+\text{d}+\gamma )}\right]+\left[\frac{\left(\text{a}+\text{b}+\text{c}+\text{d}\right)-\left(\text{a}+\text{b}\right)+\text{a}-\alpha \text{1}}{(\text{a}+\text{b}+\alpha \text{1})(\text{1}+\text{a}+\text{b}+\text{c}+\text{d}+\alpha )}\right]+\left[\frac{\left(\text{a}+\text{b}+\text{c}+\text{d}\right)-\left(\text{a}+\text{c}\right)+\beta -\beta \text{1}}{(\text{a}+\text{b}+\beta \text{1})(\text{1}+\text{a}+\text{b}+\text{c}+\text{d}+\beta )}\right]\right\}$	
	$\gamma =\gamma \text{11}\frac{\left(\text{a}+\text{b}+\text{c}+\text{d}+\alpha \mathrm{\backslash rightleft}(\text{a}+\text{b}+\text{c}+\text{d}+\beta \right)}{\left(\text{a}+\text{b}+\alpha \text{1}\right)\left(\text{a}+\text{b}+\beta \text{1}\right)}$	
	${\text{IC}*}_{\text{025}}=\text{E}\left(\text{IC}\right)-\text{1}.\text{96}\sqrt{\text{V}(\text{IC})}$	
	In which α1=β1=1; α=β=2; γ11=1	
MGPS	EBGM=$\frac{\text{a}(\text{a}+\text{b}+\text{c}+\text{d})}{(\left(\text{a}+\text{c}\mathrm{\backslash rightleft}(\text{a}+\text{b}\right))}$	EBGM05 > 2 and a > 0
	EBGM05=${\text{e}}^{\text{ln}\left(\text{EBGM}\right)-\text{1}.\text{64}(\frac{\text{1}}{\text{a}+\frac{\text{1}}{\text{b}}}+\frac{\text{1}}{\text{c}+\frac{\text{1}}{\text{d})asciicircum\text{0}.\text{5}}}}$	

Abbreviations: ROR, reporting odds ratio; PRR, proportional reported ratio; BCPNN, Bayesian confidence propagation neural network; CI, confidence interval; IC, information component; MGPS, multi-item gamma Poisson shrinker, EBGM, Empirical Bayesian Geometric Mean.

### 2.4 Statistical analysis

Continuous variables were expressed as medians with interquartile ranges (IQRs), while categorical variables were presented as frequencies and percentages. Chi-square tests were used for comparisons in large samples, and Fisher’s exact tests were applied for smaller datasets. Univariate and multivariate logistic regression models were employed to identify predictors of severe adverse events, with covariates selected based on clinical relevance. Statistical significance was defined at a two-tailed alpha level of 0.05. All analyses and visualizations were performed using Microsoft Excel (2021) and R Studio (version 2024.12.0, Build 467).

### 2.5 Ethics statement

All data used in this study were obtained from the publicly accessible U.S. Food and Drug Administration Adverse Event Reporting System (FAERS). The dataset is fully de-identified and contains no personally identifiable information. As such, this study is exempt from institutional review board (IRB) approval in accordance with relevant ethical guidelines and PLOS ONE policies.

## 3. Results

### 3.1 Clinical features of cases

**Fig 2:**
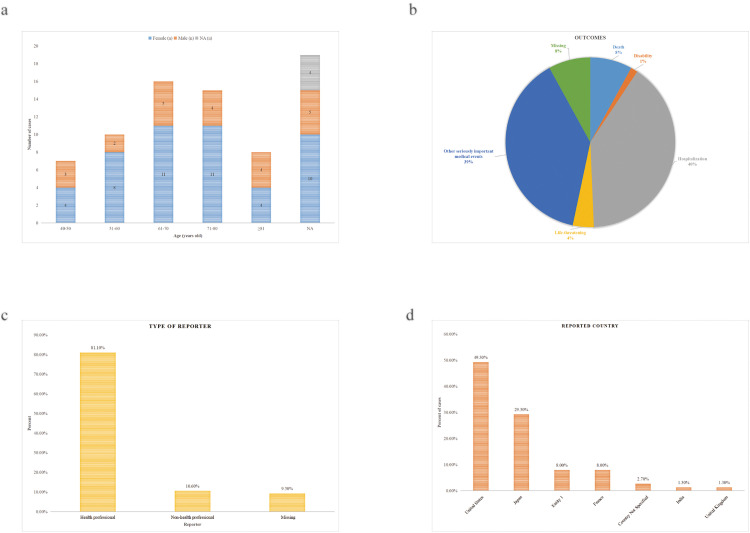
Clinical characteristics of patients. **(a)** Age and gender distribution. **(b)** Outcomes of 75 reported cases. **(c)** Reporter classification. **(d)** Distribution by country.

This study analyzed 43,958 adverse event reports related to bowel cleansers, identifying 75 cases of IC. The mean age of affected patients was 66.1 ± 12.5 years, with 64.0% being female. The average age at onset was 64.9 ± 11.8 years for females and 66.0 ± 14.0 years for males. Females outnumbered males in all age groups from 40 to 80 years. There was no significant age distribution difference between genders, either within subgroups (p = 0.335) or overall (p = 0.731). Detailed data are presented in Fig 2A, Table 3, and S3 Table.

**Table 3:** Clinical features of bowel cleansers associated cases.

Characteristics	Case number (N = 75)	Case proportion (%)
Gender		
F	48	64.00%
M	23	30.70%
Missing	4	5.30%
Weight (Kg)		
<50	2	2.70%
50 ~ 100	15	20.00%
>100	1	1.30%
Missing	57	76.00%
	Mean±SD	66.81 ± 16.1
	Median (Q1, Q3)	66 (59.42,70.65)
AGE		
<18岁	0	0%
>85岁	0	0%
18 ~ 64.9岁	26	34.70%
65 ~ 85岁	30	40.00%
Missing	19	25.30%
	Mean±SD	66.11 ± 12.54
	Median (Q1, Q3)	65 (56.75, 78)
Female	Mean±SD	64.9 ± 11.8*
	Median (Q1, Q3)	65.0 (59.5,75.0)
Male	Mean±SD	66.0 ± 14.0*
	Median (Q1, Q3)	66.0 (56.0,79.0)
	Female vs. male*	p-value = 0.731
Type of reporter		
Health professional	60	81.10%
Non-health professional	8	10.60%
Missing	7	9.30%
Outcomes		
Hospitalization-Initial or Prolonged	30	40.00%
Other serious (important medical events)	29	38.70%
Death	6	8.00%
Life-threatening	3	4%
Disability	1	1.30%
Missing	6	8.00%
Reported Country		
United States	37	49.30%
Japan	22	29.30%
Entity 1	6	8.00%
France	6	8.00%
Country Not Specified	2	2.70%
India	1	1.30%
United Kingdom	1	1.30%

For adverse event outcomes, the most common was “hospitalization - initial or prolonged” (40.0%), followed by “other serious (important medical events)” (38.7%). Fatal outcomes and life-threatening events were less frequent, occurring in 8.0% and 4.0% of cases, respectively (Fig 2B).

Most reports (81.1%) were submitted by healthcare professionals, while non-healthcare reporters (e.g., consumers or lawyers) accounted for 10.6% (Fig 2C).

Geographically, the majority of reports originated from the United States (49.3%) and Japan (29.3%), with additional cases from France, India, and the United Kingdom. A small number of reports did not specify the source country (Fig 2D).

### 3.2 Reporting trends

**Fig 3:**
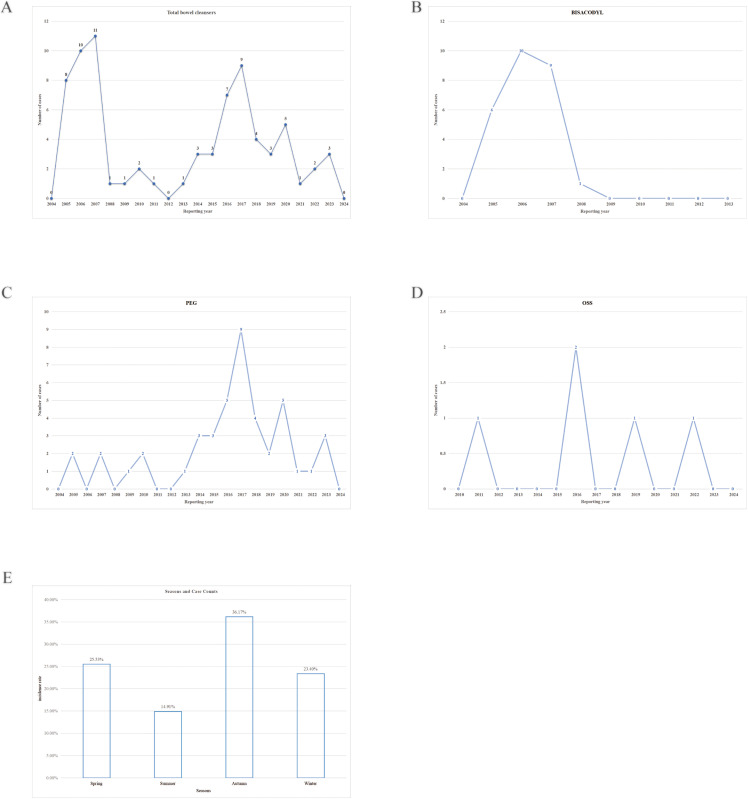
Annual case count. **(a)** Cases associated with all bowel cleansers. **(b)** Cases associated with bisacodyl. **(c)** Cases associated with PEG. **(d)** Cases associated with OSS. **(e)** Seasonal variation and Case Counts.

Since 2004, the reporting trends of IC cases showed two distinct peaks: a primary peak in 2007 and a secondary peak in 2017 (Fig 3A). Among the 75 cases, 26 were associated with bisacodyl, 44 with PEG, and 5 with OSS. Bisacodyl was the main contributor to the initial peak (Fig 3B), while the later increase was linked to PEG use (Fig 3C). Notably, bisacodyl-related reports ceased after 2009, and no OSS cases were reported before 2010. OSS cases remained sporadic (0–2 cases annually) throughout the study period (Fig 3D).

The median annual reporting proportion for all drugs was 0.13%, with bisacodyl showing the highest reporting proportion (7.90%), followed by PEG (0.07%) and OSS, which had the lowest observed reporting proportion (<0.01%). Detailed data are provided in Fig 3 and S4 Table.

Most cases originated from northern hemisphere countries, with seasonal distribution defined as spring (March–May), summer (June–August), autumn (September–November), and winter (December–February). Among the reported IC cases, 36.17% occurred in autumn, 25.5% in winter, 23.4% in spring, and 14.9% in summer. Despite these numerical differences, seasonal variation was not statistically significant (Fig 3E and S5 Table).

### 3.3 Association of medications with IC

Disproportionality analyses consistently showed a strong association between bowel cleansers and IC (ROR: 1136.7, PRR: 890.79, IC025: 9.43, EBGM05: 715.68). Among the three agents, bisacodyl exhibited the strongest link to IC, with ROR of 237.25 (95% CI: 159.72–352.44), PRR of 224.32 (95% CI: 223.95–224.7), IC (IC_025_) of 7.81 (7.24), and EBGM (EBGM05) of 223.88 (160.77) (Table 4).

**Table 4:** Disproportionality analysis.

Drug	PT	ROR (95%Cl)	PRR (95%Cl)	IC(IC_025_)	EBGM(EBGM05)
Bisacodyl	Target	237.25 (159.72 - 352.44)	224.32 (223.95-224.7)	7.81 (7.24)	223.88 (160.77)
	Non-target	0 (0 - 0.01)	0.95 (0.92-0.97)	−0.08 (−0.27)	0.95 (0.68)
PEG	Target	2.18 (1.63 - 2.93)	2.18 (1.89-2.48)	1.12 (0.7)	2.18 (1.71)
	Non-target	0.46 (0.34 - 0.61)	1 (1−1)	0(−0.01)	1 (0.78)
OSS	Target	3.64 (1.52 - 8.76)	3.64 (2.76-4.52)	1.86 (0.69)	3.64 (1.75)
	Non-target	0.27 (0.11 - 0.66)	1 (1−1)	0 (−0.06)	1 (0.48)
Total	Target	1136.7 (881.21 - 1466.26)	890.79 (890.59-890.99)	9.79 (9.43)	885.6 (715.68)
	Non-target	0 (0 − 0)	0.78 (0.73-0.84)	−0.35 (−0.58)	0.78 (0.63)

**Fig 4:**
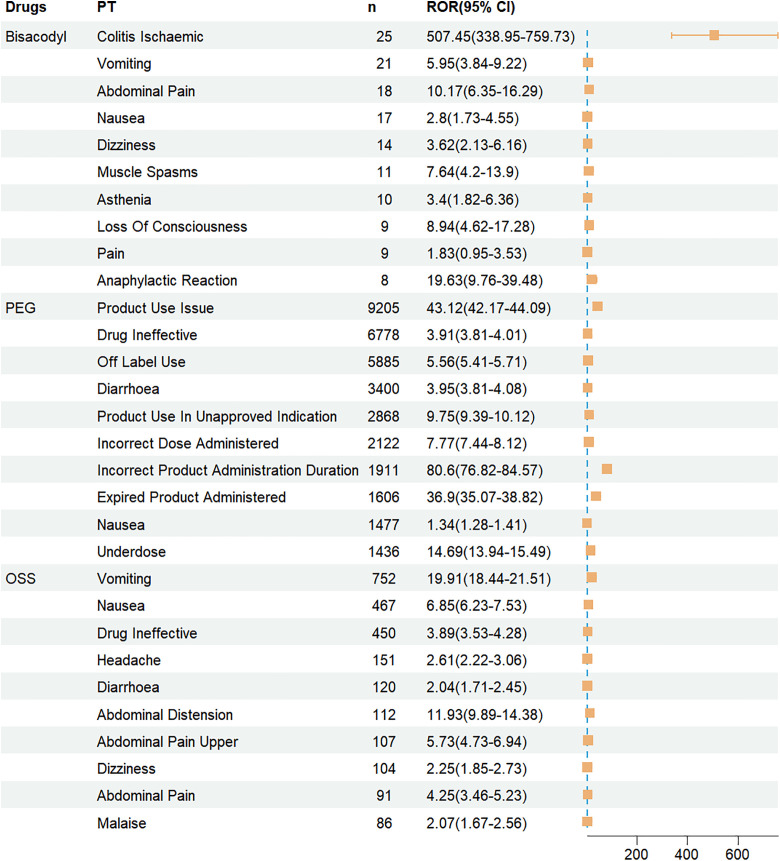
Top 10% PTs of bowel cleansers ranked by case frequency.

Further analysis of the top 10% preferred terms by case frequency revealed that bisacodyl had a significantly higher association with IC (n = 25, ROR: 507.45; 95% CI: 338.95–759.73) compared to PEG and OSS. For PEG, the most common adverse events were related to medication use issues, with “Product Use Issue” being the most frequently reported PT (n = 9205; ROR: 43.12; 95% CI: 42.17–44.09). The highest ROR for PEG was observed for “Incorrect Product Administration Duration” (n = 1911, ROR: 80.6; 95% CI: 76.82–84.57). OSS was most frequently associated with “Vomiting” (n = 752, ROR: 19.91; 95% CI: 18.44–21.51). Detailed results are shown in Fig 4.

### 3.4 Clinical indications and outcomes

As shown in Table 5, bowel cleansers were primarily used for bowel preparation and colonoscopy. Among 26 bisacodyl users, 21 (80.8%) used it for colonoscopy, with 17 receiving a 20 mg dose. Of the 44 PEG users, 23 (30.67%) underwent bowel preparation and 13 (17.34%) had colonoscopy. For OSS, 1 patient (1.3%) used it for bowel preparation and another for colonoscopy.

**Table 5:** Clinical indications and outcomes by type of bowel cleanser.

Indications	Bisacodyl n (%)	PEG n (%)	OSS n (%)	p-value
Anorexia		1 (1.33%)		
Bowel Preparation		23 (30.67%)	1 (1.33%)	
Colonoscopy	21 (28.00%)	8 (10.67%)	1 (1.33%)	
Constipation		3 (4.00%)		
Endoscopy Large Bowel		5 (6.67%)		
Irritable Bowel Syndrome		1 (1.33%)		
Laxative Supportive Care		2 (2.67%)		
Product Used for Unknown Indication			3 (4.00%)	
Missing	5 (6.67%)	1 (1.33%)		
Total (n = 75)	26 (34.67%)	44 (58.67%)	5 (6.66%)	
**Outcomes**				
Death	0 (0%)	6 (13.6%)	0 (0%)	*0.012*
Disability	0 (0%)	1 (2.3%)	0 (0%)	0.366
Hospitalization	7 (26.9%)	21 (47.7%)	2 (40.0%)	0.121
Life-threatening	0 (0%)	3 (6.8%)	0 (0%)	0.089
Other serious important medical events	13 (50.0%)	13 (29.5%)	3 (60.0%)	0.169
Required intervention to prevent permanent impairment/damage	0 (0%)	0 (0%)	0 (0%)	–
Missing	6 (23.1%)	0 (0%)	0 (0%)	–
Total (n = 75)	26 (100%)	44 (100%)	5 (100%)	
Oral dose: n (dose)	17 (20 mg); 9 (NA)	15 (Recommended dose) 29 (NA)	3 (Recommended dose) 2 (NA)	

#### 3.4.1 Outcomes by drug.

**Fig 5:**
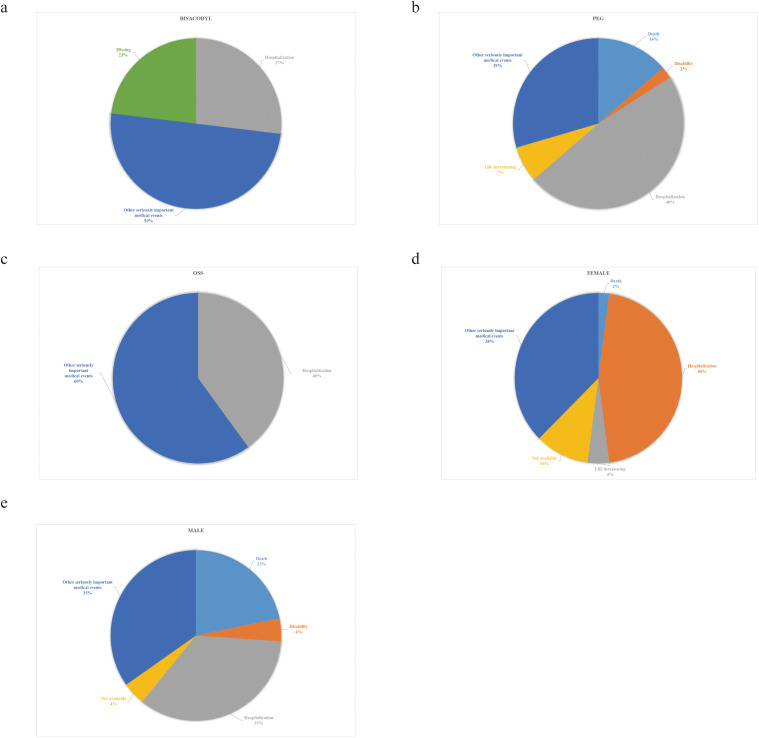
Distribution of adverse event outcomes. **(a)** Outcomes of cases using bisacodyl. **(b)** Outcomes of cases using PEG. **(c)** Outcomes of cases using OSS. **(d)** Outcomes among female patients. **(e)** Outcomes among male patients.

**Bisacodyl**: The most common outcome was “Other serious medical events” (50.0%), followed by “Hospitalization” (26.9%) (Fig 5A).

**PEG**: Nearly half required hospitalization (47.7%), with 29.5% experiencing other serious events. Notably, six patients (13.6%) died, and three (6.8%) had life-threatening complications (Fig 5B).

**OSS**: Three patients experienced serious medical events, and two required hospitalizations. No life-threatening events were reported in this group (Fig 5C).

The mortality rate was significantly higher in the PEG group compared to others (p = 0.012). No significant differences were observed among the groups for disability (p = 0.366), hospitalization (p = 0.121), life-threatening events (p = 0.089), or other serious events (p = 0.169).

#### 3.4.2 Gender differences.

Male patients had a significantly higher mortality rate than females (5/23 vs. 1/48, p = 0.012). Both genders most commonly experienced “Hospitalization” and “Other serious medical events,” with no significant differences in disability or life-threatening events. For detailed outcomes, refer to Figs 5D, 5E, and S6-7 Table.

### 3.5 Risk factors associated with IC occurrence

Univariate and multivariate logistic analyses revealed that IC was significantly associated with age and body weight but not with gender (Table 6).

**Table 6:** Logistic regression analysis of ischemic colitis occurrence.

Variable	Univariate		Multivariate	
	OR (95% CI)	P-value	OR (95% CI)	P-value
Gender				
Male vs. Female	1.2(0.95, 1.50)	0.12	1.15(0.90, 1.45)	0.25
Age (years)				
<40	1(Reference)	–	1(Reference)	–
40-49	2.5(1.80, 3.50)	<0.01	2.3(1.60, 3.30)	<0.01
50-59	3(2.20, 4.10)	<0.01	2.8(2.00, 3.90)	<0.01
60-69	3.5(2.50, 4.90)	<0.01	3.2(2.30, 4.50)	<0.01
70-79	4(2.80, 5.70)	<0.01	3.7(2.60, 5.20)	<0.01
80-89	4.5(3.20, 6.30)	<0.01	4.2(3.00, 5.90)	<0.01
≥90	5(3.50, 7.10)	<0.01	4.8(3.40, 6.80)	<0.01
Overall	1.05 (1.03, 1.07)	<0.01	1.04 (1.02, 1.06)	<0.01
Weight				
<50 kg	1(Reference)	–	1(Reference)	–
50-100 kg	1.5(1.20, 1.90)	<0.01	1.4(1.10, 1.80)	0.01
>100 kg	2(1.50, 2.70)	<0.01	1.8(1.30, 2.50)	<0.01
Overall	1.02 (1.01, 1.03)	<0.01	1.01 (1.00, 1.02)	0.02

OR:Odds Ratio;

CI: Confidence Interval

#### 3.5.1 Gender.

Although males had a 20% higher OR for IC compared to females in univariate analysis (OR = 1.20, 95% CI: 0.95–1.50, P = 0.12) and a similar trend in multivariate analysis (OR = 1.15, 95% CI: 0.90–1.45, P = 0.25), these differences were not statistically significant.

#### 3.5.2 Age and body weight.

Both analyses consistently showed significant associations between IC and age and body weight (P < 0.05). Adjusted for gender and body weight, each additional year of age increased the odds by 4% (OR = 1.04, 95% CI: 1.02–1.06, P < 0.01). Similarly, each additional kilogram of body weight corresponded to a 1% increase in IC association (OR = 1.01, 95% CI: 1.02–1.06, P = 0.02).

### 3.6 Severe outcomes and risk factors

Mortality and life-threatening events were significantly associated with age (P < 0.05), with a notable rise in severe outcomes among individuals aged 70–79.9 years, underscoring advanced age as a key association factor. In contrast, no significant associations were found between gender, body weight, and severe outcomes (Table 7).

**Table 7:** Logistic regression analysis of mortality and life-threatening outcomes.

Group		Univariate		Multivariate	
		OR (95% CI)	p-value	OR (95% CI)	p-value
Sex	male	1.4 (0.8, 2.5)	0.25	1.3 (0.7, 2.4)	0.38
	female				
Age					
	≤ 59.9	1 (reference)		1 (reference)	
	60-69.9	1.9 (1.0, 3.6)	*0.04*	1.7 (0.9, 3.2)	0.09
	70-79.9	2.8 (1.4, 5.5)	*0.003*	2.4 (1.2, 4.8)	*0.01*
	≥80	3.8 (1.8, 8.1)	*0.0003*	3.2 (1.4, 7.1)	*0.005*
Weight	<50.9	1 (reference)		1 (reference)	
	51- 99.9	1.7 (0.9, 3.4)	0.12	1.5 (0.7, 3.1)	0.32
	≥100	2.3 (1.0, 5.4)	0.05	2.0 (0.8, 4.8)	0.12

OR:Odds Ratio;

CI: Confidence Interval

### 3.7 Concomitant medications and potential comorbidities

Due to the absence of comorbidity data in the FAERS database, concurrent medication use was analyzed. Among the 75 IC patients, 138 concomitant drugs were identified (see S8 Table), with 30 (21.74%) being cardiovascular agents, including antihypertensives, anticoagulants/antiplatelets, lipid-lowering drugs, antiarrhythmics, and diuretics.

Notably, among the 9 patients who died or experienced life-threatening events, 41 medications were used concurrently, of which 16 (39.02%) were cardiovascular drugs (Table 8). These findings suggest that cardiovascular diseases may contribute to IC development, particularly in severe cases.

**Table 8:** Concomitant medications with bowel cleansers in total and severe outcome cases.

Category	Total cases		Cases with Severe Outcomes	
	Count	Percent	Count	Percent
Cardiovascular Drugs	30	21.74%	16	39.02%
Gastrointestinal Drugs	8	5.80%	6	14.63%
Antibiotics	6	4.35%	2	4.88%
Analgesics	8	5.80%	1	2.44%
Antiallergics	4	2.90%	1	2.44%
Antidepressants	6	4.35%	1	2.44%
Hormonal Drugs	7	5.07%	1	2.44%
Vitamins and Minerals	9	6.52%	0	0.00%
Urological Drugs	1	0.72%	1	2.44%
Others	12	9%	6	15%
Unknown or Unclassified	5	3.62%	0	0%
Total	138	100%	41	100%

*Cardiovascular Drugs: antihypertensives, antianginals, anticoagulants/antiplatelets, lipid-lowering agents, antiarrhythmics, vasodilators, heart failure medications, and diuretics.

*Gastrointestinal Drugs: Include antacids, antiemetics, and others.

## 4. Discussion

Ischemic colitis is a condition associated with transient or sustained reduction in colonic blood flow, leading to mucosal injury. It typically presents with sudden onset of lower abdominal pain, urgency, and hematochezia within 24 hours of bowel preparation. Mild cases resolve with supportive care, including fluid resuscitation and bowel rest, while severe cases may progress to bowel necrosis, perforation, or strictures, requiring surgical intervention. Prompt diagnosis via abdominal CT and colonoscopy is crucial for early management and prevention of complications [2,15,16].

Consistent with the literature, our study found that most patients were elderly and female, with an average age of 66.11 years and 64.00% being women. Although IC can occur in younger adults without cardiovascular comorbidities [3], no cases were found in individuals under 18. Middle-aged adults (40–64.9 years) accounted for 34.7% of cases. Age-stratified analysis showed no significant distribution difference compared to patients over 65. While female patients were slightly younger on average (64.9 vs. 66.0 years), no significant gender differences were observed across age groups.

Various factors, including medications, can cause IC [15,17]. Drugs linked to IC include diuretics, antibiotics, antihypertensives, digoxin, oral contraceptives, and NSAIDs [16]. However, only one large-scale cohort study from Japan has investigated IC related to bowel preparation agents [4]. Among 14,924 patients undergoing bowel preparation for colonoscopy from 2011 to 2020, 14 developed IC, yielding a reporting proportion of 0.09%. The regimen included magnesium citrate with 2L of PEG-ELS for non-constipated patients, while constipated patients received an additional dose of sodium picosulfate hydrate. For younger, non-hypertensive patients who struggled with PEG-ELS, 50 sodium phosphate tablets were used as an alternative. The study identified that using a potent regimen (magnesium citrate, sodium picosulfate, and PEG-ELS) in constipated patients over 75 years old was an independent association factor for IC.

A nationwide survey by Israeli physicians reported 8 cases of bisacodyl-induced IC between 2014 and 2019. All patients experienced abdominal discomfort after taking 10 mg of bisacodyl three days before the examination. Three patients continued with the same dose the following day, along with Picolax (containing 10 mg sodium picosulfate, 3.5 g magnesium oxide light, and 12 g citric acid) [6].

Current bowel cleansing regimens typically combine bisacodyl tablets with PEG electrolyte solution or oral sulfate solution, with volumes ranging from 1 to 4 liters [18,19]. Ultra-low-volume options, such as a 0.3L regimen, are also available [20], along with formulations including sodium picosulfate and sodium phosphate [19,21].

Bisacodyl tablets, commonly used as stimulant laxatives in bowel preparation, enhance the cleansing effect of PEG or OSS. The association of bisacodyl-induced IC is dose-dependent. Available in 5 mg, 10 mg, 15 mg, and 20 mg doses, the 20 mg dose is more likely to cause abdominal cramping, nausea, and IC compared to the 10 mg dose [22,23]. In turn, the 10 mg dose carries a higher IC association than the 5 mg dose. As a result, the U.S. FDA withdrew approval of the 10 mg dose for bowel preparation in 2011, leaving only the 5 mg dose on the market [24].

Our study found that the median reporting proportion of IC associated with all bowel cleansers was 0.13%, slightly higher than the figure reported in the Japanese study mentioned earlier. Among individual agents, bisacodyl had the highest median reporting proportion (7.90%), followed by PEG (0.07%) and OSS, which showed the lowest observed reporting proportion (<0.01%). The reporting proportion of IC with bisacodyl was the highest. Among the 26 cases of bisacodyl-induced IC, 17 involved the use of 20 mg of bisacodyl, further supporting the strong association between high-dose bisacodyl and IC.

A study found that bisacodyl enhances bowel cleansing only in ultra-low-volume regimens but increases discomfort in high-association patients, such as those with a history of abdominal surgery or obstipation. In contrast, high-volume (4L) or low-volume (2L) regimens generally provide satisfactory cleansing, and adding bisacodyl did not improve efficacy. Therefore, bisacodyl may be omitted in 2L or 4L regimens to reduce discomfort and the association with IC. The study did not specify the bisacodyl dosage used [20].

Reports of IC associated with PEG are rare. One case involved a patient developing symptoms after consuming 1L of PEG-ELS [5], and another described a 70-year-old woman with hyperlipidemia and hypertension who developed IC after a colonoscopy prepared with PEG, though mechanical factors from the procedure may have contributed [25,26]. PEG, particularly the isosmotic PEG-ELS introduced in the 1980s, has a well-established safety profile and few restrictions [21].

The mechanism of PEG-induced IC is unclear and may be idiosyncratic. A possible cause is fluid loss leading to dehydration and colonic hypoperfusion [5]. Our study identified 44 cases of IC associated with PEG, significantly more than previously reported. This may be due to PEG’s long history, well-established safety profile, and widespread use as a bowel preparation agent. Of the 44 cases, 23 (30.67%) were for bowel preparation, while 13 were for colonoscopy. Guidelines for bowel preparation are developed by gastroenterology societies, making gastroenterologists more aware of IC related to bowel cleansers. In contrast, surgeons and radiologists may be less familiar with PEG-induced IC, leading to underreporting and the misconception that its reporting proportion is low.

Oral sulfate solutions were approved for use in Japan and the United States around 2008–2010 [27,28]. Studies suggest they have a lower association with IC compared to other bowel preparation agents [29]. Our study supports this, finding only 5 IC cases with no reports in many years. However, OSS was introduced more recently and is less widely used than PEG, which may explain the lower number of IC cases. Vigilance is still needed for IC associated with OSS, particularly in younger patients without cardiovascular diseases [30].

An observational study found a cumulative incidence of IC of 0.02% for both OSS and PEG, with no significant difference between the two [29]. IC associated with OSS may result from both the rapid shift of intravascular fluid into the colonic lumen, causing inadequate perfusion, and the magnesium sulfate in OSS, which stimulates colonic motility via cholecystokinin and prostaglandins, potentially leading to ischemia [31].

While sodium phosphate requires less fluid intake and improves patient compliance, it poses a risk of phosphate nephropathy. Furthermore, NaP is not superior to PEG-ELS for bowel preparation, and guidelines do not recommend its routine use [18,24,32].

Advanced age and higher body weight are commonly linked to an increased incidence of cardiovascular conditions, such as hypertension and coronary heart disease [33]. In our study, age and body weight were association factors for IC, while gender had no significant impact. Severe outcomes were primarily associated with advanced age, with those aged 70 and above at higher association of bowel cleanser-induced IC and fatal complications, likely due to the higher prevalence of cardiovascular comorbidities. Additionally, patients with severe outcomes were more likely to be on cardiovascular medications.

Cold weather has been linked to increased cardiovascular events [34]. In our study, IC reports peaked in autumn (36.17%), followed by winter and spring. However, seasonal differences were not statistically significant, suggesting that bowel cleanser–associated IC is unlikely to be confounded by seasonal variation.

In summary, our findings suggest notable associations between bowel preparation agents—particularly bisacodyl—and ischemic colitis, with increased severity observed in elderly patients and those with cardiovascular comorbidities. However, this study has limitations inherent to the FAERS database, including underreporting, reporting bias, and lack of exposure data, which preclude estimation of true incidence or risk. Therefore, the associations observed should not be interpreted as causal. Prospective studies are warranted to validate these signals and better define the safety profiles of bowel preparation agents.

Additionally, the classification of combination regimens under the bisacodyl category may introduce confounding, as co-administered agents such as PEG could also contribute to adverse outcomes. In our dataset, PEG monotherapy was associated with the highest number of IC reports. Therefore, the elevated ROR observed in the bisacodyl group may be partly driven by cases involving bisacodyl-PEG co-administration. Furthermore, the combination of bisacodyl and PEG may exert additive effects in provoking ischemic colitis. As a stimulant and an osmotic laxative, respectively, their synergistic action could result in excessive colonic motility, dehydration, and transient mucosal hypoperfusion. This pharmacologic interaction may increase mucosal vulnerability and help explain the disproportionality signal observed. Further studies are warranted to assess the safety of such regimens, particularly in elderly or high-risk individuals.

## 5. Conclusion

To our knowledge, this is one of the largest pharmacovigilance analyses exploring ischemic colitis associated with bowel preparation agents. Leveraging 20 years of FAERS data, we identified strong, drug-specific disproportionality signals: bisacodyl showed the highest reporting proportion of IC among its adverse-event reports and the strongest association with severe outcomes.

While previous clinical studies have suggested a low overall association with IC, our findings raise concerns about this assumption, particularly regarding the safety of polyethylene glycol in elderly and high-association patients. The identification of bisacodyl as a major contributor to IC highlights the need for dose-dependent risk stratification in bowel preparation protocols.

Given the increasing reliance on colonoscopy for colorectal cancer screening, clinicians must reconsider bowel preparation regimens for elderly and cardiovascularly compromised patients. Future prospective studies and regulatory evaluations are needed to reassess the safety guidelines for bowel cleansers.

### Abbreviations

**Table:** 

	Introduction
IC	Ischemic colitis
FAERS	Food and Drug Administration’s Adverse Event Reporting System
PEG	Polyethylene glycol
OSS	Oral sulfate solutions
	Methods and Results
AEs	adverse events
PTs	preferred terms
SD	Standard deviation
SOC	system organ class
ROR	Reporting Odds Ratio
PRR	Proportional Reporting Ratio
BCPNN	Bayesian Confidence Propagation Neural Network
MGPS	Multi-item gamma Poisson shrinker
EBGM	Empirical Bayesian Geometric Mean
95% CI	95% confidence interval;
IC*	information component
IC*_025_	the lower limit of 95% CI of the IC
E(IC*)	the IC expectations
V(IC*)	the variance of IC
EBGM05	the lower limit of 95% CI of EBGM
OR	Odds Ratio
NA	Not available

## Supporting information

S1 Table Medications screened for target adverse events. (DOCX)

S2 Table The MedDRA code of prefer terms. (DOCX)

S3 Table Age and gender distribution analysis (Fisher’s Exact Test and Chi-Square Test). (DOCX)

S4 Table Annual case count (Based on All Cases). (DOCX)

S5 Table Seasonal distribution of cases. (DOCX)

S6 Table Clinical characteristics of IC patients caused by three types of drugs. (DOCX)

S7 Table Outcomes comparison between female and male. (DOCX)

S8 Table Concomitant medications administered with bowel cleansers. (DOCX)

S2 Data Analysis data of manuscript. (ZIP)
