# Supplementary material for: Oral bowel cleansers and ischemic colitis risk: A real-world disproportionality analysis
Source: PLoS One. 2025 Sep 29;20(9):e0332345. doi: 10.1371/journal.pone.0332345 (PMC12478960; doi:10.1371/journal.pone.0332345)
Supplement: S1 Table — (DOCX) [file pone.0332345.s001.docx]

Supplemental Table 1. Medications Screened for Target Adverse Events.

| **PEG** | |
| --- | --- |
|  | SUCLEAR, |
|  | CLENZ-LYTE, |
|  | CO-LAV, |
|  | COLOVAGE, |
|  | COLYTE, |
|  | COLYTE WITH FLAVOR PACKS, |
|  | COLYTE-FLAVORED, |
|  | E-Z-EM PREP LYTE, |
|  | GLYCOPREP, |
|  | GO-EVAC, |
|  | GOLYTELY, |
|  | LAX-LYTE WITH FLAVOR PACKS, |
|  | MOVIPREP, |
|  | NULYTELY, |
|  | NULYTELY-FLAVORED, |
|  | OCL, |
|  | PEG 3350 AND ELECTROLYTES, |
|  | PEG-3350, POTASSIUM CHLORIDE, SODIUM BICARBONATE, SODIUM CHLORIDE, |
|  | PEG-3350, SODIUM SULFATE, SODIUM CHLORIDE, POTASSIUM CHLORIDE, SODIUM ASCORBATE AND ASCORBIC ACID, |
|  | PEG-LYTE, |
|  | PLENVU, |
|  | POLYETHYLENE GLYCOL 3350, |
|  | POLYETHYLENE GLYCOL 3350 AND ELECTROLYTES, |
|  | SUFLAVE, |
|  | TRILYTE |
| **Bisacodyl** | |
|  | POLYETHYLENE GLYCOL 3350; SODIUM CHLORIDE; SODIUM BICARBONATE; POTASSIUM CHLORIDE; BISACODYL, |
|  | HALFLYTELY, |
|  | PEG-3350, SODIUM CHLORIDE, SODIUM BICARBONATE, POTASSIUM CHLORIDE AND BISACODYL, |
|  | PEG-3351, SODIUM CHLORIDE, SODIUM BICARBONATE, POTASSIUM CHLORIDE AND BISACODYL, |
| **OSS** | |
|  | COLPREP KIT, |
|  | MAGNESIUM SULFATE; POTASSIUM SULFATE; SODIUM SULFATE, |
|  | SODIUM SULFATE, POTASSIUM SULFATE AND MAGNESIUM SULFATE, |
|  | SUPREP BOWEL PREP KIT, |
|  | SUTAB, |
|  | CITRIC ACID; MAGNESIUM OXIDE; SODIUM PICOSULFATE, |
|  | CLENPIQ, |
|  | PREPOPIK, |
|  | SODIUM PICOSULFATE, MAGNESIUM OXIDE AND ANHYDROUS CITRIC ACID, |
|  | SODIUM PICOSULFATE |

PEG: polyethylene glycol

OSS: Oral sulfate solution

| Prefer term |  | MedDRA Code |
| --- | --- | --- |
| Colitis Ischaemic |  | 10009895 |
| Ischaemic Enteritis |  | 10074063 |
| Intestinal Ischaemia |  | 10022680 |
| Gastrointestinal Ischaemia |  | 10059028 |
| Visceral Arterial Ischaemia |  | 10022680 |
| Mesenteric Artery Thrombosis |  | 10027397 |
| Mesenteric Vascular Occlusion |  | 10074583 |
| Thrombosis Mesenteric Vessel |  | 10043626 |
| Mesenteric Vein Thrombosis |  | 10027402 |
| Mesenteric Arterial Occlusion |  | 10027394 |
| Mesenteric Artery Embolism |  | 10027395 |
| Mesenteric Vascular Insufficiency |  | 10027401 |
| Mesenteric Artery Stenosis |  | 10027396 |
| Mesenteric Venous Occlusion |  | 10027403 |
| Portosplenomesenteric Venous Thrombosis |  | 10077623 |
| Portosplenomesenteric Venous Thrombosis |  | 10074583 |

Supplemental table 2. The MedDRA code of Prefer terms

Supplemental table 3. Age and Gender Distribution Analysis (Fisher's Exact Test and Chi-Square Test)

| Age (years old) |  | Female (%) | Male (%) | NA (%) | Total (%) | Statistic | P-Value |
| --- | --- | --- | --- | --- | --- | --- | --- |
| 40-50 |  | 4 （57.14%） | 3 （42.86%） | 0 | 7 (100%) | 1.333 | 0.678 |
| 51-60 |  | 8 （80.00%） | 2 （20.00%） | 0 | 10 (100%) | 4 | 0.057 |
| 61-70 |  | 11 (68.75%) | 5 (31.25%) | 0 | 16 (100%) | 2.2 | 0.138 |
| 71-80 |  | 11 (73.33%) | 4 (26.67%) | 0 | 15 (100%) | 2.75 | 0.097 |
| ≥81 |  | 4 （50.00%） | 4 (50.00%) | 0 | 8 (100%) | 1 | 1 |
| NA |  | 10 (52.63%) | 5 (26.31%) | 4 (21.05%) | 19 (100%) |  |  |
| Total (n) |  | 48 (64.00%) | 23 (30.67%) | 4 (5.33%) | 75 (100%) | 4.56 | 0.335* |

* Chi-Square Test.

Supplemental table 4. Annual case count (Based on All Cases)

| **Reporting Year** | **Bisacodyl (n=26)** | **PEG (n=44)** | **OSS (n=5)** | **Total Cases (N=75)** | **All Cases (N=43958)** |
| --- | --- | --- | --- | --- | --- |
|  | Cases (%) | Cases (%) | Cases (%) | Cases (%) | All Cases* (%) |
| 2004 | - | - | - | - | 29 (100%) |
| 2005 | 6 (6.98%) | 2 (2.32%) | - | 8 (9.30%) | 86 (100%) |
| 2006 | 10 (16.13%) | 0 (0.00%) | - | 10 (16.13%) | 62 (100%) |
| 2007 | 9 (8.82%) | 2 (1.25%) | - | 11 (10.78%) | 102 (100%) |
| 2008 | 1 (0.63%) | 0 (0.00%) | - | 1 (0.63%) | 160 (100%) |
| 2009 | - | 1 (0.85%) | - | 1 (0.56%) | 177 (100%) |
| 2010 | - | 2 (3.13%) | - | 2 (1.71%) | 117 (100%) |
| 2011 | - | 0 (0.00%) | 1 (1.56%) | 1 (1.56%) | 64 (100%) |
| 2012 | - | 0 (0.00%) | 0 (0.00%) | 0 (0.00%) | 440 (100%) |
| 2013 | - | 1 (0.04%) | 0 (0.00%) | 1 (0.03%) | 3603 (100%) |
| 2014 | - | 3 (0.07%) | 0 (0.00%) | 3 (0.11%) | 2806 (100%) |
| 2015 | - | 3 (0.05%) | 0 (0.00%) | 3 (0.07%) | 4404 (100%) |
| 2016 | - | 5 (0.09%) | 2 (0.03%) | 7 (0.12%) | 5930 (100%) |
| 2017 | - | 9 (0.19%) | 0 (0.00%) | 9 (0.17%) | 5425 (100%) |
| 2018 | - | 4 (0.10%) | 0 (0.00%) | 4 (0.09%) | 4645 (100%) |
| 2019 | - | 2 (0.07%) | 1 (0.03%) | 3 (0.08%) | 3837 (100%) |
| 2020 | - | 5 (0.17%) | 0 (0.00%) | 5 (0.17%) | 3020 (100%) |
| 2021 | - | 1 (0.04%) | 0 (0.00%) | 1 (0.03%) | 2951 (100%) |
| 2022 | - | 1 (0.04%) | 1 (0.04%) | 2 (0.08%) | 2511 (100%) |
| 2023 | - | 3 (0.25%) | 0 (0.00%) | 3 (0.13%) | 2393 (100%) |
| 2024 | - | 0 (0.00%) | 0 (0.00%) | 0 (0.00%) | 1196 (100%) |
| **Median** | 7.5 (7.90%) | 2 (0.07%) | 0 (0.00%) | 3 (0.13%) | - |

*All Cases = Target Cases + Non-Target Cases.

Supplemental Table 5. Seasonal Distribution of Cases

| Reporting year | Spring | Summer | Autumn | Winter |
| --- | --- | --- | --- | --- |
|  |  |  |  |  |
| 2004 | 0 | 0 | 1 (2.13%) | 3 (6.39%) |
| 2005 | 1 (2.13%) | 3 (6.39%) | 2 (4.26%) | 2 (4.26%) |
| 2006 | 2 (4.26%) | 0 | 7 (14.91%) | 0 |
| 2007 | 1 (2.13%) | 0 | 2 (4.26%) | 0 |
| 2008 | 0 | 0 | 0 | 0 |
| 2009 | 1 (2.13%) | 0 | 0 | 0 |
| 2010 | 0 | 0 | 1 (2.13%) | 0 |
| 2011 | 1 | 0 | 0 | 0 |
| 2012 | 0 | 0 | 0 | 0 |
| 2013 | 0 | 0 | 0 | 0 |
| 2014 | 0 | 0 | 0 | 1 (2.13%) |
| 2015 | 1 (2.13%) | 0 | 3 | 1 (2.13%) |
| 2016 | 1 (2.13%) | 0 | 1 (2.13%) | 0 |
| 2017 | 3 (6.39%) | 2 (4.26%) | 0 | 1 (2.13%) |
| 2018 | 0 | 0 | 0 | 1 (2.13%) |
| 2019 | 0 | 0 | 0 | 0 |
| 2020 | 0 | 1 (2.13%) | 0 | 1 (2.13%) |
| 2021 | 0 | 1 (2.13%) | 0 | 0 |
| 2022 | 1 (2.13%) | 0 | 0 | 0 |
| 2023 | 0 | 0 | 0 | 1 (2.13%) |
| 2024 | 0 | 0 | 0 | 0 |
| Total(n=47,100%) | 12 (25.53%) | 7 (14.91%) | 17 (36.17%) | 11 (23.4%) |
| NA (n=28) |  |  |  |  |
| Chi-squared = 7.23, df = 3, p-value = 0.065 | | | | |

Data are number (%) of cases.

Supplemental table 6. Clinical Characteristics of IC Patients Caused by Three Types of Drugs

| Characteristics | Bisacodyl | PEG | OSS |
| --- | --- | --- | --- |
|  | (N=26) | (N=44) | (N=5) |
|  |  |  |  |
| SEX |  |  |  |
| Female | 18 (69.2%) | 27 (61.4%) | 3 (60.0%) |
| Male | 8 (30.8%) | 15 (34.1%) | 0 (0%) |
| Missing | 0 (0%) | 2 (4.5%) | 2 (40.0%) |
| Total | 26(100%) | 44(100%) | 5(100%) |
|  |  |  |  |
| Weight (Kg) |  |  |  |
| ＜50 | 0 (0%) | 2 (4.5%) | 0 (0%) |
| ＞100 | 1 (3.8%) | 0 (0%) | 0 (0%) |
| 50～100 | 11 (42.3%) | 3 (6.8%) | 1 (20.0%) |
| Missing | 14 (53.8%) | 39 (88.6%) | 4 (80.0%) |
| Total | 26(100%) | 44(100%) | 5(100%) |
|  |  |  |  |
| Age (years old) |  |  |  |
| <18 | 0 (0%) | 0 (0%) | 0 (0%) |
| 18～64.9 | 10 (38.5%) | 12 (27.3%) | 0 (0%) |
| 65～85 | 2 (7.7%) | 18 (40.9%) | 2 (40.0%) |
| >85 | 14 (53.8%) | 0 (0%) | 0 (0%) |
| Missing | 0 (0%) | 14 (31.8%) | 3 (60.0%) |
| Total | 26(100%) | 44(100%) | 5(100%) |

**Supplemental Table 7. Outcomes comparison between female and male.**

| Outcome | Female | Male | Statistic | p-value |
| --- | --- | --- | --- | --- |
|  |  |  |  |  |
| Death | 1(2.08%) | 5(21.74%) | - | 0.012 |
| Disability | 0(0%) | 1(4.35%) | - | 0.321 |
| Hospitalization | 22(45.83%) | 8(34.78%) | 0.89 | 0.345 |
| Life threatening | 2(4.17%) | 0(0%) | - | 0.543 |
| Not available | 5(10.42%) | 1(4.35%) | - | 0.412 |
| Other seriously important medical events | 18(37.5%) | 8(34.78%) | 0.07 | 0.791 |
|  |  |  |  |  |
| Total | 48(100%) | 23(100%) |  |  |

Data = n (%).

Supplemental table 8. Concomitant Medications Administered with Bowel Cleansers.

| Category | Drug Names |
| --- | --- |
| Cardiovascular Drugs | PLAVIX, NIFEDIPINE, ENALAPRIL MALEATE, ATORVASTATIN, CARVEDILOL, NICORANDIL, HERBESSER, XARELTO, VALSARTAN, HALFDIGOXIN, MAGNESIUM OXIDE, EDIROL, LEVOTHYROXINE, VALDECOXIB, BAYASPIRIN, HALFDIGOXIN |
| Gastrointestinal Drugs | TAKEPRON, OMEPRAZOLE, DOMPERIDONE, TAKECAB, ULCERLMIN, MAGMITT, SENNOSIDE |
| Antibiotics | PASETOCIN, KLARICID |
| Analgesics | TOFRANIL |
| Antiallergics | BETANIS |
| Antidepressants | TOFRANIL |
| Hormonal Drugs | LEVOTHYROXINE |
| Vitamins and Minerals | ASCORBIC ACID, GARLIC TABLETS SOFT GEL, FOLIC ACID, COQ10, OSCO500 D CALCIUM, MULTI VITAMIN, MULTI MINERAL, CENTRUM SILVER, CALCIUM |
| Urological Drugs | VESICARE |
| Others | ZESULAN, BICALUTAMIDE, HEMOPORISON, MIYA BM, URINORM, PARIET, EDIROL, VALDECOXIB |

*Cardiovascular Drugs: antihypertensives, antianginals, anticoagulants/antiplatelets, lipid-lowering agents, antiarrhythmics, vasodilators, heart failure medications, and diuretics.

*Gastrointestinal Drugs: Include antacids, antiemetics, and others.
